# Variability of rehabilitation protocols for ulnar collateral ligament repair with suture tape augmentation

**DOI:** 10.1016/j.xrrt.2024.07.005

**Published:** 2024-08-06

**Authors:** Benjamin M. Ose, Henry Wang, Christopher D. Bernard, Erik Mersereau, Rachel Long, Bryan G. Vopat, Erik Henkelman, Matthew L. Vopat

**Affiliations:** a University of Kansas School of Medicine, Kansas City, KS, USA; b Department of Orthopedics and Sports Medicine, University of Kansas Medical Center, Kansas City, KS, USA

**Keywords:** Ulnar collateral ligament, Repair, Augmentation, Rehabilitation, Protocol, Overhead throwing

## Abstract

**Background:**

The ulnar collateral ligament (UCL) is frequently injured in overhead throwing athletes, leading to elbow instability, pain and decreased performance, and requiring surgical intervention. Augmenting with suture tape provides a novel approach to UCL repair, offering stability while preserving native anatomy, proprioception, and minimizing bone loss, with the added benefit of an expected faster rehabilitation and return to sport (RTS) compared to traditional UCL reconstruction. The purpose of this study is to assess the variability of the current publicly available rehabilitation protocols for elbow UCL repair with suture tape augmentation.

**Methods:**

A systematic review of Google and PubMed was performed to find rehabilitation protocols for UCL repair with suture tape augmentation. Two hundred nine orthopedic surgery residency programs were identified using the Fellowship and Residency Electronic Interactive Database Access. The programs were searched on Google, a broad nonspecific Google search, and a systematic literature search of journal databases for UCL repair with suture tape augmentation rehabilitation protocols was conducted.

**Results:**

Thirteen rehabilitation protocols met the inclusion criteria for analysis. Of these, 12 protocols outlined the use of an articulating brace at varying range of motion settings for the first 4-6 weeks following surgery. Return to throwing was included in all 13 protocols and began between 10 and 12 weeks following surgery. RTS was included in 11 protocols and expected athletes competing as early as 16 weeks with a mean recommended RTS at 21.5 weeks.

**Conclusion:**

Rehabilitation protocols for UCL repair with suture tape augmentation were often structured around a 5-phase program with RTS approaching 20 weeks. They utilized immobilization and range of motion restriction as well as strengthening and gradual RTS procedures. Overall, the included protocols had mild variability with initiation of throwing and RTS 12 to 24 weeks faster than traditional UCL reconstruction.

The ulnar collateral ligament (UCL) located on the medial aspect of the elbow provides the primary restraint to valgus forces within the elbow.^13^^,^^32^ Injury to this ligament is prevalent in athletic populations, particularly among baseball pitchers, javelin throwers, and other overhead throwing athletes.^13^^,^^27^ The high-demand nature of overhead throwing places significant stress on the UCL, especially during the late cocking and early acceleration phases of throwing. Repetitive stress leads to chronic wear and thickening of the ligament, which may lead to UCL injury, or an acute traumatic event may occur resulting in UCL injury and ultimately instability or laxity of the elbow.^11^^,^^30^ UCL injuries often necessitate a period of rest, rehabilitation, and in some cases, surgical intervention, resulting in prolonged absence from competition. UCL reconstruction has historically been the surgical intervention of choice to restore elbow stability and allow athletes to return to their sport.^4^ However, UCL repair with suture tape augmentation is an emerging technique that takes a different approach.^7^ This repair utilizes a scaffold or suture tape to reinforce the repaired ligament and preserves the patients’ native anatomy and proprioception. At higher levels of tension, this can work to remove some of the stress to the ligament as well as limit bone loss due to the reconstructive procedure.^1^

Rehabilitation protocols play an important role in optimizing outcomes following a procedure. They are designed to guide the recovery process in a way that will promote tissue healing, restore range of motion (ROM), improve muscular strength and endurance, enhance neuromuscular control, and facilitate a safe return to sport (RTS). Prior studies have looked at the rehabilitation protocols for UCL reconstruction but lack the inclusion or discussion of UCL repair with suture tape augmentation.^5^^,^^13^ UCL repair using suture tape augmentation has become an accepted alternative to traditional UCL reconstruction when the ligament is still of good quality but damaged on the proximal end, distal end, or both.^7^^,^^10^^,^^18^^,^^22^^,^^31^ Repair is often cited as having the primary goals of enhancing elbow joint stability and allowing an earlier RTS timeline for athletes.^30^ The rehabilitation process following a UCL repair is different and often accelerated compared to a UCL reconstruction rehabilitation protocol. However, no study to our knowledge has reviewed and compared specifically the rehabilitation protocols following UCL repair. Therefore, the purpose of this study was to review and compare the existing rehabilitation protocols used by orthopedic surgery programs in the United States specifically for UCL repair with suture tape augmentation. The findings of this investigation may support more cohesive guidelines and highlight common elements or differences among protocols published by different programs. Compiling these protocol recommendations may additionally help to provide a comparison between UCL reconstruction and UCL repair rehabilitation.

## Materials and methods

**Figure 1:**
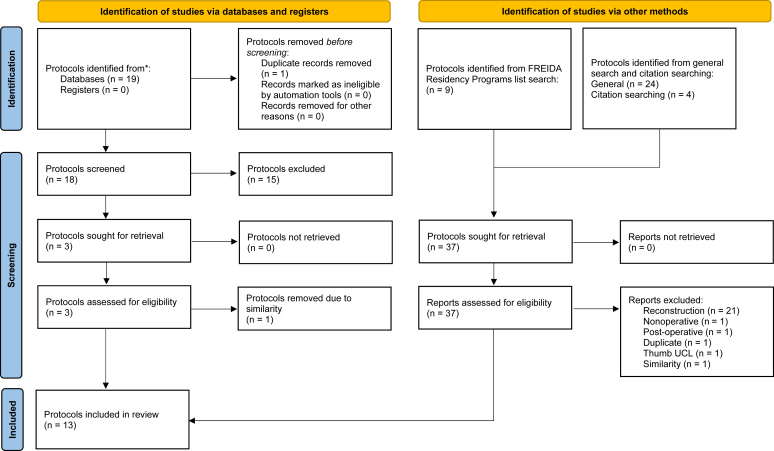
Search strategy flow chart for papers/protocols identified.

Using the Fellowship and Residency Electronic Interactive Database Access program search tool provided by the American Medical Association, a list of 209 orthopedic residency programs were identified. Each program was used in a Google search “[Program Name] UCL Repair with suture tape augmentation rehabilitation protocol” to find all publicly available protocols provided by the institution. Additionally, general and database searches were conducted. The 2 general Google searches used the phrases “UCL Repair with internal brace rehabilitation protocol” and “UCL Repair with suture tape augmentation rehabilitation protocol” and included the first 5 pages of results to encompass protocols published by private practice groups. Finally, the terms [(ulnar collateral ligament OR UCL) AND rehabilitation AND (internal brace OR suture tape augmentation)] were searched into PubMed to identify papers published in peer-reviewed journals. Review article’s citations were additionally examined for citation of rehabilitation protocols. Figure 1 illustrates this entire process in a flow diagram.

Inclusion criteria consisted of protocols that specify elbow UCL repair with internal brace or augmentation with suture tape. Exclusion criteria consisted of protocols that outlined nonoperative care, focused on postoperative recovery, lacked a timeline of rehabilitation, generalized elbow rehabilitation, focused on UCL reconstruction, or focused on thumb UCL repair.

Included protocols were analyzed for both the inclusion and timing/initiation of certain aspects of rehabilitation throughout the recovery of the patient. Each protocol was analyzed for key benchmarks along the rehabilitation timeline. These included the use of a splint and brace, passive and active elbow ROM, exercise supplementation, stretching, strengthening, hitting programs, throwing programs, and RTS. Additionally, several of these were looked at in comparison to traditional UCL reconstruction and how the timeline may be expedited using repair with suture tape augmentation.

## Results

### Description of included studies

The initial search strategy yielded 55 protocols and papers eligible for screening. Fifteen journal articles did not meet the inclusion criteria, 21 protocols focused on reconstruction or failed to specify UCL repair with suture tape augmentation, 1 focused on nonoperative rehabilitation, 1 focused primarily on postoperative care, 1 focused on thumb UCL repair, and 2 were not unique protocols. Ultimately, 13 protocols were included for review. The protocols were differentiated into peer-reviewed journal articles, protocols associated with academic universities or from physicians with academic appointments, and protocols from private practice groups. Six (46%),^7^^,^^12^^,^^19^^,^^24^^,^^30^^,^^31^ protocols were published in peer-reviewed journals, often containing additional rationale for certain aspects of their protocol. Three (23%),^9^^,^^20^^,^^26^ protocols were associated with academic universities or physicians with academic appointments and 4 (30%),^14^^,^^15^^,^^21^^,^^33^ protocols came from private practices, all of which can be seen in Appendix A. One published journal article and 1 academic protocol were nearly identical to one of the published journal articles^30^ and were therefore excluded but can still be seen in Appendix A.

### Phases of rehabilitation

Although there is variability among protocols, most phases and benchmarks remained consistent. Phase 1 is the immediate postoperative care with primary goals of healing tissue, preventing complications, and achieving full wrist ROM by first follow-up visit (day 7-10). Phase 2 is primarily done within an elbow ROM brace with gradual strengthening exercises. Progression into the next phase involves elbow ROM of 0 to 125 with minimal pain and tenderness. Phase 3 focuses on progression of upper extremity strength. Phase 4 advanced this with the introduction of throwing and phase 5 consisted of RTS considerations. The goals of these phases and criteria for progression are outlined in Table I.

**Table I:** Example phases of rehabilitation with goals for each phase and criteria for progression compiled from eight protocols.

	Phase 1	Phase 2	Phase 3	Phase 4	Phase 5
	(Weeks 0-1)	(Weeks 2-4/5)	(Weeks 4/6-8)	(Weeks 9-14)	(Weeks 14+)
	Initial phase	Acute/controlled mobility phase	Intermediate phase	Advanced phase	Return to play phase
Goals	- Tissue/repair healing - Reduce pain and inflammation - Full-wrist ROM - Minimize muscle atrophy	- Restore elbow ROM with brace - Improve muscle strength - Normalize joint arthrokinematics	- Full, nonpainful, AROM, without brace − 70% strength of affected shoulder - Full upper-extremity mobility - Coordinated kinetic chain involvement with single arm	- Advanced strength, power, and endurance training - Introduction to interval throwing program - Gradual return to throwing	- Return to competitive throwing - Continue all previous exercises
Progression criteria	- Purely time based^30^	- Elbow AROM 0-125 - Minimal pain and tenderness - Good manual muscle testing^8^^,^^21^^,^^30^	- Full nonpainful elbow, wrist, and shoulder ROM - No pain or tenderness - Isokinetic test that fulfills criteria to throw - Satisfactory clinical exam - Completion of rehab phases without difficulty ^8^^,^^15^^,^^21^^,^^29^, ^30^, ^31^^,^^33^	Criteria to begin long toss program outlined in SLU protocol: ^20^ - Full nonpainful ROM - Strength measures (ER/IR, others comparing to nonthrowing shoulder) - Satisfactory clinical exam - Satisfactory function tests: prone ball drop, one arm ball throws, single leg step downs, prone plank	- RTS pending physician decision and rehabilitation team (week 20+)

*RTS*, return to sport; *ROM*, range of motion; *AROM*, active range of motion; *ER*, external rotation; *IR*, internal rotation; *SLU*, Saint Louis University.

To provide an outline, the most common rehabilitation timeline begins with an immediate splint after surgery with transition to a hinged elbow brace upon the first follow-up visit 5-7 days postoperatively. Weeks 3-4 postoperatively typically consist of gradual ROM increases and initiation of the Thrower’s Ten Program. Once full ROM is achieved, the articulating elbow brace is removed. Weeks 4-8 consist of plyometrics, elbow strengthening, and continuation or progression of the Thrower’s Ten program. Weeks 8-10 fortifies previous exercises with gradual resistance training incorporating weight machine exercises. By week 12, an interval throwing program is introduced with gradual return to mound throwing by week 14-16. Typically, the RTS recommendation is 6+ months with the earliest RTS at 14 weeks depending on physician recommendation.

### Summary of outcomes assessed

The 3 primary variables of protocols analyzed included: activities within the first 6 weeks postoperatively, the variability between the initiation of an interval throwing program, and the RTS timeline. Each rehabilitation protocol was further analyzed using the major landmarks along the rehabilitation timeline which can be seen in Table II. These included the use of splints or bracing for stability and limiting ROM, the initiation of passive and active ROM, shoulder and elbow strengthening, the inclusion of the Thrower’s Ten program and progression of plyometric exercises. Finally, we assessed when athletes could begin hitting and throwing, and at what time athletes could RTS. These were each important indicators in a player's progression from surgery back to the field with many requiring certain standards to be met before progressing into the next phase of rehabilitation.

**Table II:** Number of protocols that included each rehabilitation component and mean time in weeks of beginning.

Rehabilitation component	Protocols		Average timeline
	n	(%)	(weeks)
Splint or Brace Use	12	(92%)	5.6
Passive ROM	12	(92%)	1.4
Active ROM	9	(69%)	2.0
Full ROM	9	(69%)	4.3
Shoulder Strengthening	7	(54%)	1.9
Elbow Strengthening	8	(62%)	4.6
Thrower’s Ten Program	10	(77%)	3.4
Advanced Thrower's Ten Program	8	(62%)	5.0
2-Hand Plyometrics	9	(69%)	6.8
1-Hand Plyometrics	9	(69%)	8.3
Interval Hitting	8	(62%)	10.1
Interval Throwing	13	(100%)	11.4
Return to Sport	11	(85%)	21.5

*ROM*, range of motion.

### Splint and brace use

The use of a splint immediately postoperatively and an articulating brace during rehabilitation is an important aspect of the UCL repair recovery process. These tools help restrict movement and can be adjusted to increase ROM gradually as the patient begins to increase the use of their repaired ligament. Eleven (85%)^7^^,^^9^^,^^15^^,^^19^, ^20^, ^21^^,^^24^^,^^26^^,^^30^^,^^31^^,^^33^ protocols recommended the use of an articulating brace at some point during rehabilitation. Only 1 (8%)^14^ did not recommend the use of an articulating brace after the removal of a splint at 7 days and 1 (8%)^12^ did not explicitly mention any bracing at all.

During the first week following surgery, 6 (46%)^14^^,^^15^^,^^21^^,^^24^^,^^26^^,^^33^ rehabilitation protocols recommended the use of a solid splint. In addition, 5 (38%)^7^^,^^9^^,^^20^^,^^30^^,^^31^ protocols recommended a hinged elbow brace locked for the first 7 days with the brace set to 70° or 90° flexion with no passive or active ROM. In the second week of rehab or around day 8 following surgery, 7 (54%)^15^^,^^20^^,^^21^^,^^30^^,^^31^^,^^33^ of the protocols recommended that the articulating brace be unlocked between 30° and 110° flexion. In the third week of following surgery, 8 (62%)^15^^,^^20^^,^^21^^,^^26^^,^^30^^,^^31^^,^^33^ protocols recommended the brace ROM be increased to 10° to 125° flexion. In the fourth week following surgery, 7 (54%)^15^^,^^21^^,^^26^^,^^30^^,^^31^^,^^33^ of the protocols recommended that the brace ROM be increased to between 0° and 145° flexion, with 1 protocol recommending unrestricted movement.^20^ Only 1 protocol recommended discontinuation of the brace after week 4,^9^ with the majority (38%)^20^^,^^21^^,^^30^^,^^31^^,^^33^ of the protocols removing the brace after week 6.

### Passive and active elbow ROM

Of the protocols included for review, 7 (54%)^7^^,^^9^^,^^15^^,^^21^^,^^26^^,^^33^ recommended that passive ROM start during the first week following surgery while 5 (38%)^14^^,^^20^^,^^24^^,^^30^^,^^31^ recommended starting at the beginning of week 2. Active elbow ROM was recommended at similar time points. One protocol^33^ had patients starting active ROM in the first week following surgery while 7 (54%)^9^^,^^15^^,^^20^^,^^21^^,^^24^^,^^30^^,^^31^ protocols recommended starting after day 6-10 or the beginning of the second week. Full elbow ROM was a component of 9 (69%)^7^^,^^9^^,^^14^^,^^15^^,^^19^^,^^21^^,^^24^^,^^30^^,^^31^ protocols and a requirement for progression into the next phase. Nine (69%)^7^^,^^9^^,^^14^^,^^15^^,^^19^^,^^21^^,^^24^^,^^30^^,^^31^ protocols recommended that full ROM be reached in week 4 of rehabilitation, and of these there were 3 (23%)^14^^,^^19^^,^^21^ protocols extending that to a range between 4 and 6 weeks.

### Elbow strengthening

Elbow strengthening exercises were incorporated in 8 (62%)^7^^,^^12^^,^^19^^,^^24^^,^^26^^,^^30^^,^^31^^,^^33^ protocols with variability in the timeline of initiation of these exercises. Exercises typically included wrist stretching, flexion, and forearm pronation and supination with gradual manual resistance. One (8%)^30^ protocol recommended elbow exercises starting as early as week 3 of rehabilitation with one starting isotonic exercises at 10 days postoperation.^7^ Four (31%)^12^^,^^26^^,^^31^^,^^33^ protocols recommended starting in week 4, once full ROM had been established. Finally, 2 (15%)^19^^,^^24^ protocols suggested a more conservative route to beginning elbow strengthening. While light upper extremity strengthening exercises were recommended week 1 following surgery, isolated elbow strengthening exercises began in week 6.

### Shoulder strengthening

Shoulder strengthening was incorporated into 7 (54%)^9^^,^^12^^,^^15^^,^^20^^,^^21^^,^^30^^,^^31^ rehabilitation protocols. Initiation of these exercises varied between protocols and the exercises followed a pattern of isometric movements to begin followed by external rotation muscle building. Four (31%)^15^^,^^20^^,^^30^^,^^31^ protocols recommended these early isometric strengthening exercises in the first week of recovery, while 3 (23%)^9^^,^^12^^,^^21^ recommended waiting until weeks 2 to 4. Two (15%)^21^^,^^30^ of the protocols recommended initiation of further shoulder strengthening between the weeks 4-8 and focusing on external rotation and building muscle. While these shoulder specific rehabilitation components were included, it is worth noting that the Thrower’s Ten program was also often mentioned as a method for building back shoulder strength.

### Weight machines

With the goal of advancing strength, power, and endurance as well as neuromuscular control, the initiation of weight machine exercises for resistance training was a common component of 8 (62%)^12^^,^^14^^,^^20^^,^^21^^,^^26^^,^^30^^,^^31^^,^^33^ rehabilitation protocols. Of these, all 8 (62%) began the use of weight machine exercises during week 10 of the protocol. These exercises included the use of chest press, rows, or latissimus dorsi pull-downs in varying combinations or alone.

### Thrower's Ten program

The Thrower’s Ten exercise program is a series of exercises outlined in Wilk et al^28^ and aims to structure more aggressive isotonic strengthening movements while also emphasizing the recuperation of muscle balance. The specific movements in this program are outlined in Table III. The use of this program in the rehabilitation of patients with a UCL repair with suture tape augmentation procedure was included in 10 (77%)^7^^,^^9^^,^^12^^,^^15^^,^^20^^,^^21^^,^^26^^,^^30^^,^^31^^,^^33^ protocols and the progression to the advanced Throwers Ten program was included in 8 (62%).^7^^,^^15^^,^^20^^,^^21^^,^^26^^,^^30^^,^^31^^,^^33^ Seven (54%)^7^^,^^15^^,^^20^^,^^26^^,^^30^^,^^31^^,^^33^ protocols began introducing the Thrower’s Ten program at the start of the third week or around day 15 postoperatively. Only 3 (23%)^9^^,^^12^^,^^21^ recommended waiting until week 4 or later to begin these exercises.

**Table III:** The Thrower’s Ten and advanced thrower’s ten program exercise lists.^20^^,^^28^

Thrower’s Ten program	Advanced Thrower’s Ten program
Diagonal pattern D2 extension	External rotation at 0° abduction while seated on a stability ball
Diagonal pattern D2 flexion	Internal rotation at 0° abduction while seated on a stability ball
External rotation at 0° abduction	External rotation at 0° abduction with sustained hold while seated on a stability ball
Internal rotation at 0° abduction	Internal rotation at 0° abduction with sustained hold while seated on a stability ball
Shoulder abduction to 90°	Shoulder abduction to 90° with sustained hold while seated on a stability ball
Scaption, external rotation (“full cans”)	Scaption, external rotation (“full can”) with sustained hold while seated on a stability ball
Side-lying external rotation	Side-lying external rotation
Prone horizontal abduction	Prone horizontal abduction with sustained hold on stability ball
Prone horizontal abduction (full external rotation, 100° abduction)	Prone horizontal abduction (full external rotation, 100° abduction) with sustained hold on a stability ball
Prone rowing	Prone row on a stability ball
Prone rowing into external rotation	Prone row into external rotation with sustained hold on a stability ball
Press-ups	Seated scapular retraction into external rotation on a stability ball
Push-ups	Seated low trap on a stability ball
Elbow flexion	Seated neuromuscular control on a stability ball
Elbow extension	Tilt-board push-ups
Wrist extension	Elbow flexion on a stability ball
Wrist flexion	Elbow extension on a stability ball
Wrist supination	Wrist extension
Wrist pronation	Wrist flexion
	Wrist supination
	Wrist pronation

Of the protocols investigated, 4 (31%)^7^^,^^26^^,^^31^^,^^33^ recommended the advanced Thrower’s Ten program be started in week 4, while 4 (31%)^15^^,^^20^^,^^21^^,^^30^ recommended beginning in week 6. It is important to note that progression to the advanced movements should only follow the successful completion and comfortability of the original Thrower’s Ten program.

### Plyometrics

Plyometrics are another widely used category of exercises that gradually increase functional stress to the repaired tendon and aim to improve dynamic stability and proprioception. A majority of rehabilitation protocols (9, 69%)^7^^,^^12^^,^^14^^,^^15^^,^^20^^,^^21^^,^^30^^,^^31^^,^^33^ began with 2-hand plyometric drills such as a weighted chest pass with plyometric balls. Of these, 7 (54%)^7^^,^^15^^,^^20^^,^^21^^,^^30^^,^^31^^,^^33^ recommended these drills start in week 6 of recovery while 2 (15%)^12^^,^^14^ recommended week 7-10 starting points. Upon the successful completion of 2-hand plyometric exercises most protocols progressed to 1-hand plyometrics, such as throwing into a wall, while one protocol skipped 2-handed plyometrics going straight to 1-handed. This recommended progression occurred in week 8 for 8 (62%)^7^^,^^15^^,^^20^^,^^21^^,^^26^^,^^30^^,^^31^^,^^33^ protocols and in week 10-12 for one (8%)^14^ other protocol.

### Sport specific components—interval hitting

UCL repair with suture tape augmentation is often a procedure done to overhead throwing athletes such as baseball and softball players. In these athletes, an interval hitting program is started in their rehabilitation program before a throwing program. Eight (62%)^7^^,^^12^^,^^15^^,^^20^^,^^21^^,^^30^^,^^31^^,^^33^ protocols included interval hitting programs, 7 (54%)^7^^,^^12^^,^^20^^,^^21^^,^^30^^,^^31^^,^^33^ of which began in week 10 while 1 (8%)^15^ began in week 11.

### Sport specific components—interval throwing

In our investigation all 13 (100%) protocols included the timing of an interval throwing program. One (8%)^19^ of the protocols began this program at 10 weeks, 6 (46%)^7^^,^^12^^,^^15^^,^^20^^,^^26^^,^^33^ protocols began throwing at 11 weeks and 6 (46%)^9^^,^^14^^,^^21^^,^^24^^,^^30^^,^^31^ protocols began an interval throwing program at 12 weeks or 4 months following surgery. Of these, 6 (46%)^7^^,^^12^^,^^15^^,^^21^^,^^26^^,^^33^ included the progression of the throwing program from long toss to mound throwing. In these, mound throwing began at 16 weeks in 5 (38%)^12^^,^^15^^,^^21^^,^^26^^,^^33^ protocols and after 20 weeks in 1 (8%).^7^

### Sport specific components—RTS

In our analysis, 11 (85%) rehabilitation protocols had recommendations for RTS timing. This was recommended after the successful completion of the interval throwing program, included previously mentioned exercises, and followed the recommendation of each patient's personal physician. Within the protocols, RTS began as early as weeks 16+ or after 4 months in 3 (23%) protocols.^15^^,^^19^^,^^33^ Most (7, 54%)^8^^,^^20^^,^^21^^,^^26^^,^^29^, ^30^, ^31^ protocols recommended RTS after 20+ weeks or 5 months, and 3 (23%)^9^^,^^14^^,^^24^ recommended at least 6 months of rehabilitation before return. There was often a range depending on individual athlete progression. The average RTS timeline was 21.2 weeks among these protocols.

### Stretching and mobilizations

Two less discussed and included parts of rehabilitation were stretching and the use of mobilizations. Two rehabilitation protocols,^20^^,^^30^ had the unique recommendations of grade I and II mobilizations of the elbow, while avoiding valgus stress, during the first week following surgery and going forward as needed. Both of these papers additionally recommended long duration stretching if a patient is having difficulty regaining elbow extension. This would consist of 15-minute sessions for up to 60 minutes a day of assisted stretching of the elbow using an elastic band.

## Discussion

This systematic review depicts the variability and similarities noted among rehabilitation protocols following UCL repair with suture tape augmentation. Thirteen protocols were identified in the search, with only one directly from an academic institution with an orthopedic surgery program^20^ and two others from physicians with academic appointments.^9^^,^^26^ This was fewer than what was expected and comparably fewer than other published reviews for UCL reconstruction. Additionally, while most protocols generalized repair with suture tape procedures, Scillia et al presented a novel technique building on repair with suture tape augmentation and combining it with UCL reconstruction using allograft to provide the collagen needed for longevity.^19^ This was included in our analysis because of its use of the suture tape and accelerated recovery time.

The goal of accelerated recovery time that is gained with this procedure is based around the idea that the collagen-treated suture tape augments the ligament while it is healing combined with the lack of surgical tunnels needed and no requirement of graft healing and maturation.^23^^,^^30^ While the recovery time of most protocols had players back to their sport within 20 weeks, the components of rehabilitation did not range far from that of reconstruction. It was still clear that early splint and brace use for the first week to 6 weeks is recommended as well as elbow and shoulder strengthening. While not a novel addition, the incorporation of the Thrower’s Ten program provides athletes with movements that build back strength and proprioception that they will be needed to compete. The advanced Thrower’s Ten program is a progression that adds endurance and new movements that challenge the shoulder girdle neuromuscular control as well as the rotator cuff musculature. This is achieved through alternating dynamic movements with sustained holds and can be seen in Table III. Additionally, the use of plyometric exercises support this with dynamic movements designed to systematically progress the stress the repaired ligament can undergo.

Finally, as an injury that is quite specific to overhead throwing athletes in baseball or track and field javelin throwers, the incorporation of a return to throwing and RTS are key to addressing this patient population. The throwing program is initiated to gradually reintroduce the overhand throwing motion back into the movement that likely caused their original injury. Athletes progress towards a long toss phase which progressively extends the distance that a player can throw before moving into a mound throwing phase and eventually RTS. Timing of these programs, while slightly varied, were among the most common components and in the end were impacted by the patient progression and physician comfortability.

UCL reconstruction has historically been the primary treatment option for patients with UCL insufficiency. UCL repair is indicated for certain UCL injuries including patients with sustaining a proximal or distal avulsion tear, undamaged ligaments or good-quality tissue and those with no evidence of chronic disease.^22^^,^^23^ Biomechanical studies have shown that UCL repair with suture tape augmentation has as good or lower load to failure characteristics when compared with reconstruction techniques, comparable valgus stability to the native ligament and no significant difference between reconstruction for gapping or valgus opening angle during cyclic loading.^3^^,^^25^ Other studies on contact mechanisms found no significant difference from reconstruction or the returned joint resistance, while repair had advantages in restoring joint torque.^17^ The biomechanical comparison between traditional reconstruction and repair with suture tape augmentation have supported its applicability to clinical practice.^2^^,^^3^^,^^6^^,^^10^^,^^16^^,^^17^^,^^25^

Prior studies have investigated rehabilitation following UCL reconstruction which provide a good comparison as a standard of care alternative. General themes of the postoperative rehabilitation for UCL reconstruction were similar, assessing ROM, strengthening and proprioception, plyometrics, return to throwing and RTS. Lightsey et al conducted a review of rehabilitation variability after UCL reconstruction which included aspects of 20 protocols, a mix of Electronic Residency Application Service and published works.^13^ Their review found that splinting was recommended for an average of 2.0 weeks with full ROM at 5.5 weeks and functional braces being worn through an average of 5.8 weeks.^13^ Our analysis of UCL repair found splint use recommended for an average of 6.6 days, full ROM achieved at 4.5 weeks and brace use for 5.8 weeks. While a slightly accelerated timeline for ROM for the arm, a similar timeline of brace use may suggest that the early phases of rehabilitation and healing are treated similarly between the two procedures.

As the patient progresses in their recovery, Lightsey et al found that UCL reconstruction protocols began the Throwers Ten exercise program at 7.2 weeks on average and 1-hand plyometric exercises at 14.3 weeks.^13^ This is in comparison to UCL repair initiation of the Throwers Ten exercise programs at 3.4 weeks on average and 1-hand plyometrics at 8.3 weeks. Additionally, the initiation of an interval throwing program was a key progression point in an overhead throwing athlete’s recovery. UCL reconstruction recovery protocols on average began the first phase of an interval throwing program at 16.3 weeks^13^ while UCL repair protocols started this at 11.4 weeks, nearly 5 weeks earlier during rehabilitation.

RTS is the final goal of many rehabilitation protocols for injuries closely linked to a sports related injury. Kelmer et al conducted a review investigating rehabilitation and RTS criteria following UCL reconstruction. They found that among 22 different studies, RTS was between 6.5 and 16 months with an average of 10.4 months (roughly 45 weeks).^11^ The Lightsey et al review of UCL reconstruction rehabilitation found that among 20 protocols, the average RTS was 32.9 weeks.^13^ Another study by Cheema and colleagues found a large variability in RTS following UCL reconstruction with a mean of 28 weeks.^5^ In our analysis of UCL repair, RTS was averaged at just 21.5 weeks, less than half the time of UCL reconstruction determined by Kelmer et al and 11.7 weeks faster than the UCL reconstruction timeline found by Lightsey et al.

### Limitations

This review was noted to have some limitations. First, the protocols do not outline expectations for delays. It can be expected that not all patients will progress at the same rate. The inclusion of expectations of delays or expectations of returning to sports at the same level as before injury could be beneficial. Additionally, private practice protocols were identified using a simple Google search. This search strategy has the potential to miss protocols published online by private practice groups that may not have appeared in our search. Finally, it is possible many academic programs have UCL repair with suture tape augmentation protocols that are not publicly available, which would have not appeared in the search. It is our recommendation that all academic institutions and outcome studies publish their rehabilitation protocols online for the benefit of patients recovering from surgical procedures.

## Conclusion

Rehabilitation protocols for UCL repair with suture tape augmentation were often structured around a 5-phase program with RTS around 20 weeks. They utilized immobilization and ROM restriction as well as strengthening and gradual RTS procedures. Overall, the included protocols had mild variability with initiation of throwing and RTS 12 to 24 weeks faster than traditional UCL reconstruction.

## Disclaimers:

Funding: This study did not receive any outside funding or grants.

Conflicts of interest: The authors, their immediate families, and any research foundations with which they are affiliated have not received any financial payments or other benefits from any commercial entity related to the subject of this article.
