# Supplementary material for: Variability of rehabilitation protocols for ulnar collateral ligament repair with suture tape augmentation
Source: JSES Rev Rep Tech. 2024 Aug 6;4(4):703–9. doi: 10.1016/j.xrrt.2024.07.005 (PMC11514088; doi:10.1016/j.xrrt.2024.07.005)
Supplement: Appendix A [file mmc1.docx]

**Appendix A.** Protocols included in analysis along with two protocols excluded from analysis due to similarity.

| Protocol Author | Affiliation |
| --- | --- |
| Wilk et al. (JOSPT, 2019) ^30^ | Published journal article |
| Wilk et al. (IJSPT, 2022) ^31^ | Published journal article |
| Uchida et al. ^24^ | Published journal article |
| Scilia et al. ^19^ | Published journal article |
| Dugas et al.^7^ | Published journal article |
| Kerzner et al. ^12^ | Published journal article |
| Brian Waterman, MD ^26^ | Academic - Wake Forest |
| Saint Louis University ^20^ | Academic - Saint Louis University |
| Rothman Orthopedics ^9^ | Academic - Sidney Kimmel Medical College |
| Daniel Myer, MD ^14^ | Private - Crystal Clinic Orthopedic Center |
| Corey A. Wulf, MD ^33^ | Private - Twin Cities Orthopedics |
| Jacob Stirton, MD ^21^ | Private - Union General Health System |
| Andrew Parker, MD ^15^ | Private - Texas Health Orthopedic Specialists |
| Protocols excluded due to similarity | |
| Wilk et al. (OSMOAJ, 2019) ^29^ | Published journal article |
| Josef Eichinger, MD ^8^ | Academic - Medical University of South Carolina |

JOSPT = Journal of Orthopaedic & Sports Physical Therapy

IJSPT = International Journal of Sports Physical Therapy

OSMOAJ = Orthopedics and Sports Medicine: Open Access Journal
